# Supplementary material for: TAF-ChIP: an ultra-low input approach for genome-wide chromatin immunoprecipitation assay
Source: Life Sci Alliance. 2019 Jul 22;2(4):e201900318. doi: 10.26508/lsa.201900318 (PMC6653780; doi:10.26508/lsa.201900318)
Supplement: Supplementary file 1 [file LSA-2019-00318_Supplemental_Data_1.docx]

**Detailed Protocol:**

1. Fix the Cells for 10 min at room temperature with 1% formaldehyde in PBS.
2. Quench the crosslink with 125 mM glycine, for 5 minutes at RT. Note: If the material is sufficient then wash once with ice cold PBS, else immediately proceed to cell sorting.
3. Sort cells on BD FACSAria™ cell sorter using Hoechst stain, directly into 200μl of RIPA 140 mM. Sorting small number of cells (100 cells normally comes in < 2μl) will have no effect on buffer composition owing to small volume.
4. Sonicate the cells collected in RIPA for 3 cycles at low power settings (Diagenode Bioruptor XL) for breaking the nuclei, 30 Secs “ON”/ “OFF”.
5. In the meantime, bind the 1μg of antibodies to 15μl of protein G dynabeads in 200μl of blocking buffer. Incubate the bead-ab mixture at 4°C for 2-3 hours
6. Centrifuge the sonicated cells at 2000g for 10 min at 4°C. Note: This step can be omitted.
7. Separate the antibodies bound to the beads by putting the tube on magnetic rack and remove the blocking buffer.
8. Add the supernatant from step 6 to the tube containing Ab-coupled beads.
9. Incubate the samples at 4°C overnight with head over tail rotations.
10. Separate the beads and wash the beads twice by 300μl of home-made tagmentation buffer by resuspending the beads by pipetting up and down with 1000 μl pipette. Quickly spin the tube with mini centrifuge and separate the beads by putting on magnetic rack, remove as much liquid as possible without disturbing the beads.
11. Resuspend the beads in 20 μl of 1X tagmentation buffer containing 1 μl of tagmentase, with 20 μl pipette.
12. Incubate the tubes at 37 °C for 40 min with constant shaking in a thermoblock at 500 rpm.
13. Wash the beads; once with 140 mM RIPA, four times with 250 mM RIPA and twice with TE buffer pH 8.0. Each wash should be for 3 minutes with 200 μl of wash buffer and should be performed in cold-room.
14. Resuspend the beads after washing in 50 μl of TE buffer containing 5 μl of proteinase K and incubate overnight/at-least 6 hours at 60 °C in thermoblock with shaking at 500 rpm. Either proceed to Section A or Section B.

Section A

1. Add 50 μl of TE buffer and 300 μl of Phenol:Chloroform. Vortex and centrifuge at maximum speed for 5 minutes at room temperature.
2. Transfer the upper aqueous phase and add 20 μl of 3M sodium acetate (pH 5.2), 5 μl of 20 mg/ml glycogen. Add 700 μl of 100 % ethanol. Incubate overnight at -80 °C to precipitate.
3. Centrifuge at full speed (14000 rpm) at 4 °C for 30 minutes, wash the pellet once with 75% ethanol and resuspend it in 30 μl of TE buffer.
4. Set up the PCR reaction as following;

Forward primer 20 μM (provided in table1): 2 μl

Reverse primer 20 μM (provided in table1): 2 μl

Reverse-crosslinked sample from step 17: 30 μl

Nuclease free water: 16 μl

2X NEBNext High-Fidelity PCR Mix: 50 μl

1. Set up the PCR cycling condition as following;

72°C for 3 min

{98°C for 10 sec

63°C for 30 sec

72°C for 30 sec}, 12 cycles

72°C for 5 min

Hold at 4°C.

1. Add 0.2X of Ampure XP beads, incubate for 5 minutes at room temperature to bind large fragments. Keep the supernatant.
2. Add 0.8X of Ampure XP beads to the supernatant from step17, incubate for 5 minutes to bind library fragments.
3. Wash the beads twice with 200 μl of freshly made 80% ethanol, for 30 seconds each.
4. Elute the library in 10 μl of TE-buffer. Run the sample on Agilent Bioanalyzer following manufacturer’s instructions.
5. Pool the finished libraries in equimolar amounts and sequence on Illumina platform.

Section B

16. Set up the PCR reaction as following;

Forward primer 20 μM (provided in table1): 2 μl

Reverse primer 20 μM (provided in table1): 2 μl

Reverse-crosslinked sample from step 15: 46 μl

2X NEBNext High-Fidelity PCR Mix: 50 μl

1. Set up the PCR cycling condition as following;

72°C for 3 min

95°C for 5 min

{98°C for 10 sec

63°C for 30 sec

72°C for 30 sec}, 12 cycles

72°C for 5 min

Hold at 4°C.

1. Add 0.2X of Ampure XP beads, incubate for 5 minutes at room temperature to bind large fragments. Keep the supernatant.
2. Add 0.8X of Ampure XP beads to the supernatant from step17, incubate for 5 minutes to bind library fragments.
3. Wash the beads twice with 200 μl of freshly made 80% ethanol, for 30 seconds each.
4. Elute the library in 10 μl of TE-buffer. Run the sample on Agilent Bioanalyzer following manufacturer’s instructions.
5. Pool the finished libraries in equimolar amounts and sequence on Illumina platform.

**RIPA (140 mM) RIPA (250 mM) TE Buffer**

10 mM Tris-Cl pH 8.0 10 mM Tris-Cl pH 8.0 10 mM Tris-Cl pH 8.0

140 mM NaCl 250 mM NaCl 0.5 mM EDTA pH 8.0

0.5 mM EDTA pH 8.0 0.5 mM EDTA pH 8.0

1% Triton X-100 1% Triton X-100

0.1% SDS 0.1% SDS

**Tagmentation Buffer (Home-made) Blocking Buffer**

20 mM Tris(hydroxymethyl)aminomethane pH 7.6 10 mM Tris-Cl pH 8.0

10 mM MgCl2 140 mM NaCl

20% (vol/vol) dimethyl formamide 0.5 mM EDTA pH 8.0

1% Triton X-100

0.1% SDS

0.2 mg/ml BSA

0.05 mg/ml Glycogen

0.2 mg/ml Yeast tRNA
